# Nutritional jejunostomy in esophagectomy for cancer, a national register-based cohort study of associations with postoperative outcomes and survival

**DOI:** 10.1007/s00423-020-02037-0

**Published:** 2020-11-24

**Authors:** Anders Holmén, Masaru Hayami, Eva Szabo, Ioannis Rouvelas, Thorhallur Agustsson, Fredrik Klevebro

**Affiliations:** 1 Department of Clinical Science, Intervention and Technology (CLINTEC), Karolinska Institutet, Stockholm, Sweden; 2 Department of Surgery, Södersjukhuset, Stockholm, Sweden; 3 Department of Upper Abdominal Cancer, Karolinska University Hospital, Stockholm, Sweden; 4 Department of Surgery, Örebro University Hospital, Örebro, Sweden; 5 School of Medical Sciences, Örebro University, Örebro, Sweden; 6 Department of Clinical Science and Education, Karolinska Institutet | Södersjukhuset, Stockholm, Sweden

**Keywords:** Esophageal cancer, Esophagectomy, Postoperative complications, Anastomotic leak, Feeding jejunostomy

## Abstract

**Purpose:**

Insertion of a nutritional jejunostomy in conjunction with esophagectomy is performed with the intention to decrease the risk for postoperative malnutrition and improve recovery without adding significant catheter-related complications. However, previous research has shown no clear benefit and there is currently no consensus of practice.

**Methods:**

All patients treated with esophagectomy due to cancer during the period 2006–2017 reported in the Swedish National Register for Esophageal and Gastric Cancer were included in this register-based cohort study from a national database. Patients were stratified into two groups: esophagectomy alone and esophagectomy with jejunostomy.

**Results:**

A total of 847 patients (45.27%) had no jejunostomy inserted while 1024 patients (54.73%) were treated with jejunostomy. The groups were comparable, but some differences were seen in histological tumor type and tumor stage between the groups. No significant differences in length of hospital stay, postoperative surgical complications, Clavien-Dindo score, or 90-day mortality rate were seen. There was no evidence of increased risk for significant jejunostomy-related complications. Patients in the jejunostomy group with anastomotic leaks had a statistically significant lower risk for severe morbidity defined as Clavien-Dindo score ≥ IIIb (adjusted odds ratio 0.19, 95% CI: 0.04–0.94, *P* = 0.041) compared to patients with anastomotic leaks and no jejunostomy.

**Conclusion:**

A nutritional jejunostomy is a safe method for early postoperative enteral nutrition which might decrease the risk for severe outcomes in patients with anastomotic leaks. Nutritional jejunostomy should be considered for patients undergoing curative intended surgery for esophageal and gastro-esophageal junction cancer.

## Introduction

Postoperative complications after esophagectomy are common [1] and associated with increased mortality [2–4] and long-term decreased health-related quality of life (HRQOL) [5, 6].

Esophagogastric anastomotic leak is the major complication after esophagectomy, with a reported incidence of 4–35% [1, 7, 8]. Patients who suffer an anastomotic leak have an almost tenfold increase in the 30-day mortality rate from 2–3% to 17–35% [2]. Anastomotic leaks are associated with a nutritional deficit, which can make recovery difficult.

Nutrition is fundamental for the successful treatment of esophageal cancer where malnutrition, weight loss, and cancer cachexia are particularly prevalent [9]. Early postoperative enteral nutrition has proven to be clearly beneficial and is a key component of the ERAS protocol [10]. Enteral nutrition is associated with improved levels of gut oxygenation, lower costs, and reduced postoperative length of stay compared with total parenteral nutrition [11–13].

Insertion of a nutritional jejunostomy is made with the intent to secure a nutritional route, should postoperative oral feeding be contraindicated or insufficient. The jejunostomy can be used to give full enteral nutrition after surgery or for nutritional support in combination with early oral feeding depending on the applied clinical pathway. There is a risk for catheter-related complications, most of which are minor, such as local skin contamination, dislocation, catheter site infection, and occlusion [14]. Severe complications such as small bowel necrosis and intestinal torsion are rare but potentially life-threatening [15–17].

The aim of this study was to determine if the insertion of a nutritional jejunostomy in conjunction with esophagectomy for cancer was associated with decreased postoperative morbidity. The secondary outcome was to evaluate if the opportunity to give enteral nutrition with the use of a jejunostomy was associated with improved outcomes for patients with the postoperative anastomotic leak.

## Methods

### Study design

A nationwide, retrospective, population-based cohort study from a prospectively collected national database including all patients undergoing esophageal cancer surgery in Sweden between 2006 and 2017 was performed. Data was collected from the Swedish National Register for Esophageal and Gastric Cancer, in which all patients with esophageal or gastro-esophageal cancer in Sweden are included. The register has a national coverage of 95.5% and an overall accuracy of 91% [18]. The clinical data include patient and tumor characteristics, treatment details regarding oncological and surgical management (including the insertion of a jejunostomy or not), and study outcomes.

### Exposure

Study exposure was the insertion of nutritional jejunostomy in conjunction with esophagectomy for cancer of the esophagus or the gastro-esophageal junction.

### Outcomes

All clinical data were collected from the register. Enrolled patients were cross-matched with the National Cause of Death Register via the individual unique personal identification number assigned to all Swedish residents [19]. Outcomes included overall postoperative complications stratified by surgical or non-surgical complications, with surgical complications defined as follows: Postoperative leakage was confirmed with CT scan, with an oral water-soluble contrast medium, or verified with endoscopy. Conduit necrosis was defined as confirmed ischemia of the conduit with perforation or ulcer. Bleeding was defined as blood loss of more than 2 L or need of surgical re-intervention. Chylothorax was defined as a leak that required drainage for more than 7 days or a need for surgical re-intervention. Recurrent nerve paralysis was confirmed by an otorhinolaryngologist. Abdominal or thoracic abscesses were reported when the size of the abscess exceeded 3 × 3 cm and was verified radiologically or surgically.

Included among the non-surgical complications were cardiac arrhythmias requiring medical treatment, myocardial infarction, and cerebral embolism. Pulmonary embolism was defined as radiographically confirmed embolus requiring treatment. The definition of respiratory failure was when patients required invasive or non-invasive ventilation. Pneumonia was defined as x-ray-confirmed infiltration combined with fever, cough, and/or dyspnea and infections non-related to the operation field. Septicemia was defined as a body temperature above 38.3 °C (101 °F) or below 36 °C (96.8 °F) with a positive blood culture. Length of hospital stay in days and overall all-cause mortality were calculated based on data from the National Cause of Death Register.

### Statistical methods

Multivariable logistic regression modeling, chi-square test, and Fisher’s exact test were used for binomial outcomes. The multivariable logistic regression model and the Cox proportional hazard model were pre-specified and included tumor histology, clinical tumor stage, tumor location, and ASA score. Complete case analysis was performed in the multivariable-adjusted model. The categorizations of the variables are displayed in Table 1. The Cox proportional hazard model was used for the survival analyses. The proportional hazard assumptions were tested in all models using the Grambsch and Therneau test based on Schoenfeld residuals, which did not show any violations. For each outcome, we report the odds ratio (OR) and 95% confidence interval (CI). The significance level was set at 0.05. Analyses were performed using STATA® version 13 software (StataCorp LP, College Station, Texas, USA).

**Table 1:** Baseline characteristics of patients undergoing esophagectomy for esophageal or gastro-esophageal junction cancer, stratified by nutritional jejunostomy

*n* (%)	No jejunostomy	Jejunostomy	*P* value
Total	847 (45.3)	1024 (54.7)	
Age, median (range)	66 (20–93)	66 (29–88)	0.999
Gender			0.763
Male	671 (79.2)	817 (79.8)	
Female	176 (20.8)	207 (20.2)	
Mean body weight in kg (range)	80.8 (47–141)	81.3 (51–137)	0.999
Performance status			0.104
0	452 (57.1)	598 (61.9)	
1	291 (36.8)	322 (33.3)	
2	48 (6.1)	46 (4.8)	
Unknown	56	58	
ASA score			0.458
I	292 (35.9)	347 (35.6)	
II	397 (48.8)	475 (48.7)	
III	116 (14.3)	150 (15.4)	
IV	8 (1.0)	4 (0.4)	
Unknown	34	48	
Baseline dysphagia score			0.765
No dysphagia	76 (32.8)	64 (30.9)	
Dysphagia to solid food	108 (46.6)	103 (49.8)	
Dysphagia to semi-solid food	33 (14.2)	30 (14.5)	
Dysphagia to liquids	14 (6.0)	8 (3.9)	
Total dysphagia	1 (0.4)	2 (1.0)	
Unknown	615	817	

## Results

### Patient demographics and baseline characteristics

Out of the 1871 patients who underwent surgery for esophageal or gastro-esophageal junction cancer, 847 (45.3%) were treated with no jejunostomy and 1024 (54.7%) with nutritional jejunostomy. The groups were similar with regard to age, gender, body weight, performance status, ASA score, and dysphagia score (Table 1).

### Tumor characteristics and treatment details

Jejunostomies were less frequently inserted in cT1 tumors (12.5% vs. 7.0%) and slightly more often in T2 and T3 tumors (25.4% vs. 29.1%, 46.5% vs. 48.1%, *P* = 0.002). No significant differences were found concerning clinical N-stage, tumor location, preoperative treatment, or surgical approach. Jejunostomy was more often used with a transthoracic and transhiatal approach compared to gastrectomy (*P* = 0.001, Table 2). Histological tumor type was different between the groups (*P* = 0.005), with a higher tumor burden in the jejunostomy group. There was no significant difference in neoadjuvant treatment. Jejunostomies were inserted with open technique in 852 (83.2%) patients and laparoscopic technique in 172 patients (16.8%, Table 3). The register had some missing data concerning clinical T-stage (219 patients, 11.7%), N-stage (92 patients, 4.9%), tumor location (220 patients, 11.8%), surgical technique (98 patients, 5.2%), and histological tumor type (24 patients, 1.3%) (Table 2).

**Table 2:** Tumor characteristics and treatment details of patients undergoing esophagectomy for esophageal or gastro-esophageal junction cancer, stratified by nutritional jejunostomy

*n* (%)	No jejunostomy	Jejunostomy	*P* value
Clinical T-stage			0.002
T1	106 (12.5)	72 (7.0)	
T2	215 (25.4)	298 (29.1)	
T3	394 (46.5)	493 (48.1)	
T4	35 (4.1)	39 (3.8)	
TX	97 (11.5)	122 (11.9)	
Clinical N-stage			0.653
N-negative	452 (53.4)	568 (55.5)	
N-positive	353 (41.7)	406 (39.7)	
NX	42 (5.0)	50 (4.9)	
Tumor location			0.154
Proximal	21 (2.5)	24 (2.4)	
Middle	69 (8.2)	115 (11.4)	
Distal	467 (55.6)	575 (56.9)	
GE junction	181 (21.6)	199 (19.7)	
Unknown	109	111	
Preoperative treatment			0.646
Surgery alone	342 (40.4)	443 (43.3)	
Neoadjuvant chemotherapy	146 (17.2)	175 (17.1)	
Neoadjuvant chemoradiotherapy	345 (40.7)	385 (37.6)	
Definitive chemoradiotherapy	14 (1.7)	21 (2.1)	
Surgical technique			0.001
Transthoracic esophagectomy	661 (85.0)	886 (89.1)	
Transhiatal esophagectomy	30 (3.9)	46 (4.6)	
Gastrectomy	87 (11.2)	63 (6.3)	
Unspecified	69	29	
Surgical approach			0.981
Open esophagectomy	696 (82.2)	841 (82.1)	
Minimally invasive esophagectomy	151 (17.8)	183 (17.9)	
Anastomosis level			0.582
Thoracic	749 (88.4)	897 (87.6)	
Cervical	98 (11.6)	127 (12.4)	
Histological tumor type			0.005
Adenocarcinoma	620 (73.7)	753 (74.9)	
Squamous cell carcinoma	150 (17.8)	204 (20.3)	
Other	71 (8.4)	49 (4.9)	
Unknown	6	18

**Table 3:** Postoperative complications after esophagectomy for cancer, stratified by nutritional jejunostomy

*n* (%)	No jejunostomy	Jejunostomy	*P* value
Postoperative complication	330 (39.0)	454 (44.4)	0.019
Surgical complication	223 (26.3)	282 (27.5)	0.557
Anastomotic leak	78 (10.5)	115 (12.6)	0.195
Gastric conduit necrosis	16 (1.9)	31 (3.0)	0.117
Postoperative bleeding	17 (2.0)	15 (1.5)	0.368
Chylothorax	26 (3.1)	26 (2.5)	0.487
Thoracic abscess	21 (2.5)	39 (3.8)	0.104
Abdominal abscess	11 (1.3)	13 (1.3)	0.955
Re-operation for any cause	27 (3.2)	30 (2.9)	0.746
Recurrent laryngeal nerve paralysis	21 (2.5)	44 (4.3)	0.033
Non-surgical complication	188 (22.2)	305 (29.8)	< 0.001
Cardiovascular complication	39 (4.6)	66 (6.5)	0.085
Pulmonary embolism	16 (1.9)	31 (3.0)	0.117
Pneumonia	61 (7.2)	121 (11.8)	0.001
Septicemia	38 (4.5)	66 (6.5)	0.066
Clavien-Dindo score			0.163
I	46 (18.5)	59 (18.2)	
II	74 (29.7)	110 (34.0)	
IIIa	46 (18.5)	35 (10.8)	
IIIb	47 (18.9)	56 (17.3)	
IVa	23 (9.2)	40 (12.4)	
IVb	6 (2.4)	10 (3.1)	
V	7 (2.8)	14 (4.3)	
Unknown	81	130	
Clavien-Dindo score ≥ IIIb	83 (33.3)	120 (37.0)	0.358
Median length of hospital stay in days (IQR)	15 (10–23)	16 (12–24)	0.032
30-day mortality	14 (1.7)	22 (2.2)	0.437
90-day mortality	58 (6.9)	52 (5.1)	0.105
	Open jejunostomy	Laparoscopic jejunostomy	
Total	852 (83.2)	172 (16.8)	
Postoperative complication	378 (44.4)	76 (44.2)	0.965
Surgical complication	228 (26.8)	282 (27.5)	0.557
Re-operation for any cause	26 (3.1)	4 (2.3)	0.607
Abdominal abscess	11 (1.3)	2 (1.2)	0.891
Clavien-Dindo score ≥ IIIb	79 (38.2)	41 (35.0)	0.576

### Short-term clinical outcomes and survival

Postoperative complications were reported in 330 (39.0%) patients with no jejunostomy and in 454 (44.4%) patients with jejunostomy (*P* = 0.019). No significant differences in severity of complications according to the Clavien-Dindo scoring system were observed. Missing data concerning Clavien-Dindo score was reported in 211/784 (26.9%) of the patients with complication. The median length of hospital stay was similar. No significant differences were seen concerning the incidence of anastomotic leak, gastric conduit necrosis, re-operations, or occurrences of thoracic abscesses. Recurrent laryngeal nerve paralysis was more frequently reported in patients with jejunostomy (2.5% vs 4.3%, *P* = 0.033) as were non-surgical complications (22.2% vs 29.8%, *P* < 0.001) such as pneumonia (7.2% and 11.8%, *P* = 0.001). The postoperative 90-day mortality was 6.9% in patients without jejunostomy and 5.1% in the jejunostomy group (*P* = 0.105, Table 3).

No significant differences were observed comparing open to laparoscopic jejunostomy concerning postoperative complications, surgical complications, re-operations, or Clavien-Dindo score ≥ IIIb (Table 3).

**Fig. 1:**
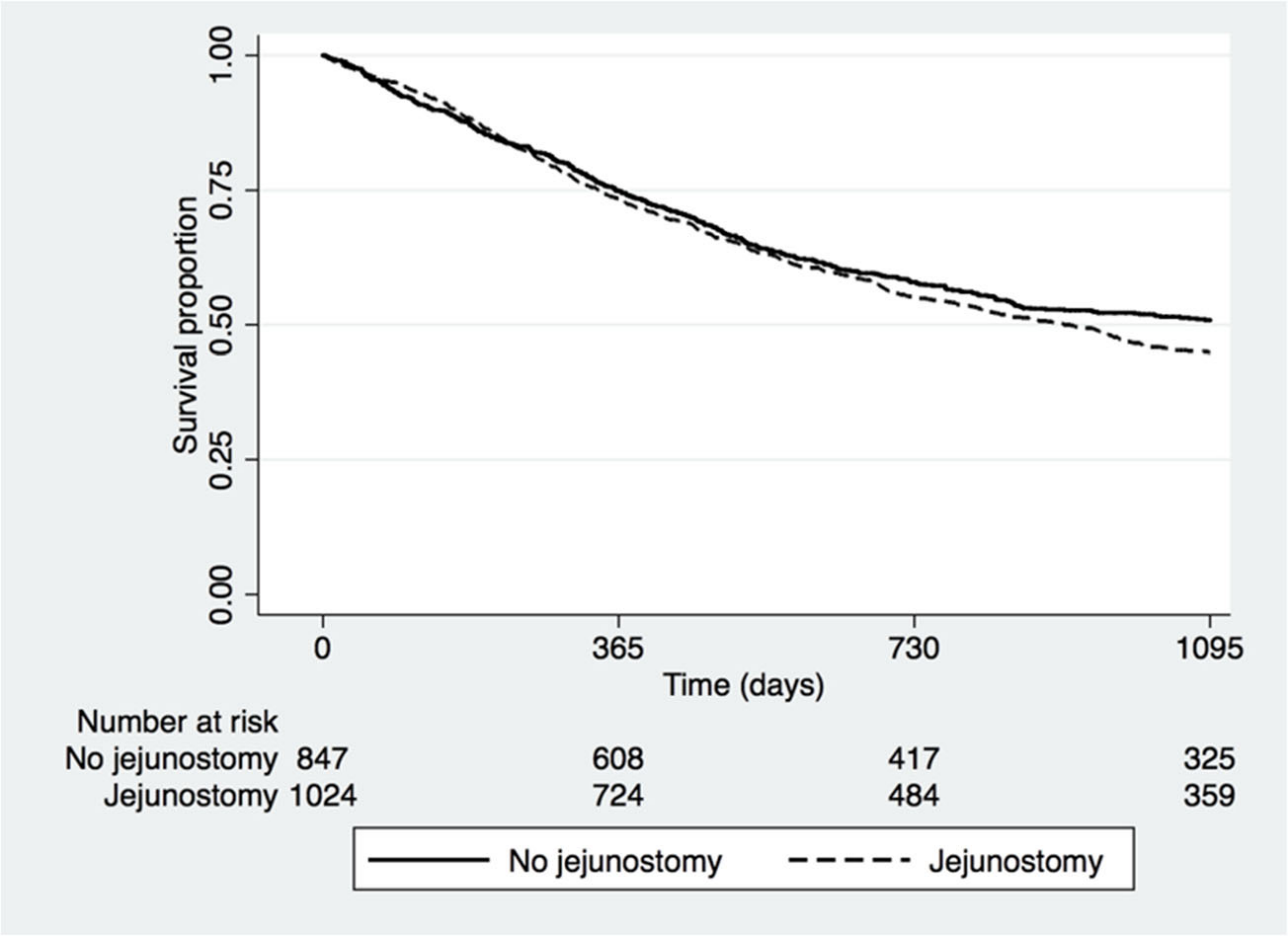
Kaplan-Meier survival curve stratified by esophagectomy with or without nutritional jejunostomy for patients treated for esophageal or gastro-esophageal junction cancer (*P* = 0.417)

Multivariable adjusted analyses showed a statistically significant increase in risk for postoperative non-surgical complications, pneumonia, septicemia, and recurrent nerve paralysis in the jejunostomy group (Table 4). There was no significant difference in long-term survival between the groups (Fig. 1).

**Table 4:** Multivariable adjusted logistic regression of postoperative complications comparing patients with jejunostomy vs. no jejunostomy in patients after esophagectomy for cancer

	No jejunostomy	Jejunostomy	*P* value
	Odds ratio (95% confidence interval)^†^		
Any complication	1.0 (reference)	1.28 (1.06–1.55)	0.011
Surgical complication	1.0 (reference)	1.07 (0.87–1.33)	0.506
Anastomotic leak	1.0 (reference)	1.21 (0.89–1.65)	0.226
Chylothorax	1.0 (reference)	0.83 (0.48–1.44)	0.507
Recurrent laryngeal nerve paralysis	1.0 (reference)	1.94 (1.11–3.38)	0.020
Non-surgical complication	1.0 (reference)	1.53 (1.23–1.90)	< 0.001
Cardiovascular complication	1.0 (reference)	1.40 (0.92–2.14)	0.117
Pulmonary embolism	1.0 (reference)	1.72 (0.92–3.21)	0.089
Pneumonia	1.0 (reference)	1.79 (1.29–2.48)	0.001
Septicemia	1.0 (reference)	1.54 (1.01–2.34)	0.043
Clavien-Dindo ≥ IIIb	1.0 (reference)	1.16 (0.81–1.65)	0.423

†Adjusted for histological tumor type, clinical tumor stage, and American Society of Anesthesiologists Score

### Clavien-Dindo score ≥ IIIb and 90-day mortality in patients with complications

Any postoperative complication within 90 days was evaluated with adjusted logistic regression analysis to assess the impact on risk for Clavien-Dindo score ≥ IIIb, as well as 90-day postoperative mortality in patients with complications comparing the no jejunostomy group to the jejunostomy group. In patients with postoperative anastomotic leakage, the odds ratio for Clavien-Dindo score ≥ IIIb was 0.19 (95% CI: 0.04–0.94) compared to patients without jejunostomy. There were no significant differences in the odds ratios for Clavien-Dindo score ≥ IIIb concerning other postoperative complications or 90-day mortality. In patients with anastomotic leakage, the odds ratio for 90-day mortality in the jejunostomy group was 0.53 (95% CI: 0.24–1.19) compared to patients with no jejunostomy. For patients with postoperative chylothorax, the corresponding odds ratio was 0.21 (95% CI: 0.03–1.33, Table 5).

**Table 5:** Risk for Clavien-Dindo score ≥ IIIb and 90-day mortality comparing patients with jejunostomy vs. no jejunostomy in patients with postoperative complications after esophagectomy for cancer

	No jejunostomy	Jejunostomy	*P* value
	Odds ratio (95% confidence interval)^†^		
Clavien-Dindo score ≥ IIIb			
Any complication	1.0 (reference)	1.09 (0.72–1.65)	0.689
Surgical complication	1.0 (reference)	1.07 (0.62–1.85)	0.815
Anastomotic leak	1.0 (reference)	0.19 (0.04–0.94)	0.041
Chylothorax	1.0 (reference)	0.67 (0.10–4.51)	0.682
Recurrent laryngeal nerve paralysis	1.0 (reference)	1.48 (0.29–7.51)	0.633
Non-surgical complication	1.0 (reference)	1.56 (0.92–2.63)	0.099
Cardiovascular complication	1.0 (reference)	2.24 (0.66–7.57)	0.196
Pulmonary embolism	1.0 (reference)	0.80 (0.15–4.12)	0.788
Pneumonia	1.0 (reference)	1.64 (0.71–3.79)	0.248
Septicemia	1.0 (reference)	0.82 (0.16–4.30)	0.817
90-day mortality			
Any complication	1.0 (reference)	0.85 (0.53–1.36)	0.498
Surgical complication	1.0 (reference)	0.71 (0.40–1.25)	0.238
Anastomotic leak	1.0 (reference)	0.53 (0.24–1.19)	0.125
Chylothorax	1.0 (reference)	0.21 (0.03–1.33)	0.098
Recurrent nerve paralysis	1.0 (reference)	0.59 (0.03–13.49)	0.740
Non-surgical complication	1.0 (reference)	0.76 (0.44–1.29)	0.309
Cardiovascular complication	1.0 (reference)	0.95 (0.31–2.88)	0.922
Pulmonary embolism	1.0 (reference)	0.86 (0.13–5.87)	0.877
Pneumonia	1.0 (reference)	0.68 (0.26–1.78)	0.431
Septicemia	1.0 (reference)	0.74 (0.26–2.09)	0.572

†Adjusted for histological tumor type, clinical tumor stage, and American Society of Anesthesiologists Score

## Discussion

The results of this population-based cohort study demonstrate that the insertion of a feeding jejunostomy in conjunction with esophagectomy was not associated with an overall increased risk for postoperative surgical complications. However, non-surgical complications, such as pneumonia and septicemia, were more common in the jejunostomy group which might be explained by confounding factors such as surgical technique and increased use of jejunostomy for patients with more locally advanced tumors. The study shows that jejunostomy in patients with the anastomotic leak was associated with a significantly lower risk for Clavien-Dindo score ≥ IIIb, which suggests that jejunostomy in conjunction with esophagectomy might increase the chance to recover from an anastomotic leak without re-operation and intensive care. A jejunostomy provides a secure route for enteral nutrition in the event of an anastomotic leak which might explain the observed improved outcome in the jejunostomy group.

The observed higher incidence of pneumonia in the jejunostomy group is, to our knowledge, not previously demonstrated and is contradictory to results shown elsewhere [20, 21]. Although it might reflect the association between jejunostomy and small bowel obstruction, as has been suggested by Koterazawa et al. [22], this was not seen in our study. Among surgical complications, the only statistically significant finding was an increased frequency of recurrent laryngeal nerve paralysis in the jejunostomy group. While this is unlikely to be related to the insertion of a jejunostomy, it is worth to notice. The increased incidence of pneumonia observed in the jejunostomy group might be explained by the higher incidence of recurrent laryngeal nerve paralysis in the jejunostomy group in terms of higher risk for aspiration. The results show a selection bias towards jejunostomy in more frail patients with a higher incidence of squamous cell carcinoma and a more advanced tumor stage. These patients require a more extensive lymph node dissection in the upper mediastinum which might explain the higher incidence of recurrent nerve palsy. The increased risk for non-surgical complications may also be explained by the increased use of jejunostomies in patients with more advanced tumor stages and in patients with the preoperative nutritional deficit, something that should be further assessed in future studies. Factors concerning baseline characteristics and type of surgery were included in the multivariable-adjusted model but there is a risk for residual confounding.

Data concerning preoperative nutritional status such as BMI and weight loss is not included in the register. High-quality data concerning weight loss is hard to evaluate since no measurements are recorded before the diagnosis. Secondary measurements concerning nutritional status such as mean body weight and baseline dysphagia score are however registered and were similar between the two groups. From the accessible data, preoperative nutritional status seems to have a minor impact on the decision to provide patients with a jejunostomy; local protocols are likely to have a more important role. It is however worth considering the risk for selection bias in the study.

Perioperative management including postoperative nutritional details or information about nasogastric tubes or early feeding is unfortunately not included in the register data. This study has analyzed the effects of the insertion of a jejunostomy at the time of the esophagectomy. A future study with more detailed data about oral, enteral, and parenteral nutrition after surgery is planned within our group.

Jejunostomy treatment details such as duration of catheter placement, degree of jejunostomy utilization, and minor jejunostomy-related complications would have been valuable to analyze, but this level of granularity of data is unfortunately not recorded in the register. However, no difference in surgical complications, Clavien-Dindo score, or re-operation was observed between the groups, which indicates that jejunostomy was not associated with increased risk for significant postoperative surgical complications.

Clavien-Dindo score was included in the register from 2012, and consequently, 211/784 (26.9%) of the patients with complication had missing data concerning Clavien-Dindo score. This is a weakness of the study; however, it is likely that this is proportionally distributed randomly between the groups. Missing data concerning tumor stage, surgical technique, and histological tumor type was taken into consideration as a complete case analysis was performed in the multivariable-adjusted model.

Strengths of the study include the population-based design, a relatively large cohort with a near-complete national coverage of all patients who underwent surgical resection for esophageal cancer in Sweden during the study period, small numbers of missing data, and the complete follow-up concerning survival made possible by the use of the National Cause of Death Register [19].

Previous research has shown that nutritional jejunostomies as part of curative treatment of esophageal cancer are safe, but controversy exists on the practice of routinely doing so, as evidence of its benefits in general is lacking [20, 23]. The nutritional deficit, weight loss, and sarcopenia are major issues that require intervention for patients undergoing esophageal cancer treatment. Jejunostomy insertion before the start of neoadjuvant treatment might provide an even more efficient nutritional treatment in selected patients [21, 24]. It is, however, challenging to design high-quality studies about nutritional treatments. Future studies need to focus on identifying patients who may benefit most from a nutritional jejunostomy, timing of placement, how and when it should be used, and also monitor changes in body composition prior to and during multimodality treatment, preferably in a randomized design.

In conclusion, this study indicates that a nutritional jejunostomy might decrease the risk for severe outcomes in patients with postoperative anastomotic leak after esophagectomy. However, our data suggests no clear benefit to apply standardized nutritional jejunostomy to all esophagectomy patients. Future research needs to investigate the optimal use of nutritional jejunostomy in esophageal cancer treatment.

## References

[1] Low DE, Kuppusamy MK, Alderson D, Cecconello I, Chang AC, Darling G, Davies A, D’Journo XB, Gisbertz SS, Griffin SM, Hardwick R, Hoelscher A, Hofstetter W, Jobe B, Kitagawa Y, Law S, Mariette C, Maynard N, Morse CR, Nafteux P, Pera M, Pramesh CS, Puig S, Reynolds JV, Schroeder W, Smithers M, Wijnhoven BPL (2019). Benchmarking complications associated with esophagectomy. Ann Surg.

[2] Evans RPT, Singh P, Nepogodiev D, Bundred J, Kamarajah S, Jefferies B, Siaw-Acheampong K, Wanigasooriya K, McKay S, Mohamed I, Whitehouse T, Alderson D, Gossage J, van Hillegersberg R, Vohra RS, Griffiths EA (2019) Study protocol for a multicenter prospective cohort study on esophagogastric anastomoses and anastomotic leak (the Oesophago-Gastric Anastomosis Audit/OGAA). Dis Esophagus Off J Int Soc Dis Esophagus. 10.1093/dote/doz007 10.1093/dote/doz007 30888419

[3] Markar S, Gronnier C, Duhamel A, Mabrut JY, Bail JP, Carrere N, Lefevre JH, Brigand C, Vaillant JC, Adham M, Msika S, Demartines N, Nakadi IE, Meunier B, Collet D, Mariette C, FREGAT (French Eso-Gastric Tumors) working group, FRENCH (Fédération de Recherche EN CHirurgie), and AFC (Association Française de Chirurgie) (2015). The impact of severe anastomotic leak on long-term survival and cancer recurrence after surgical resection for esophageal malignancy. Ann Surg.

[4] Schieman C, Wigle DA, Deschamps C, Nichols III FC, Cassivi SD, Shen KR, Allen MS (2012). Patterns of operative mortality following esophagectomy. Dis Esophagus Off J Int Soc Dis Esophagus.

[5] Derogar M, Orsini N, Sadr-Azodi O, Lagergren P (2012). Influence of major postoperative complications on health-related quality of life among long-term survivors of esophageal cancer surgery. J Clin Oncol Off J Am Soc Clin Oncol.

[6] Kauppila J, Johar A, Lagergren P (2018). Postoperative complications and health-related quality of life 10 years after esophageal cancer surgery. Ann Surg Publish Ahead of Print.

[7] Markar SR, Schmidt H, Kunz S, Bodnar A, Hubka M, Low DE (2014). Evolution of standardized clinical pathways: refining multidisciplinary care and process to improve outcomes of the surgical treatment of esophageal cancer. J Gastrointest Surg Off J Soc Surg Aliment Tract.

[8] Blencowe NS, Strong S, McNair AGK (2012). Reporting of short-term clinical outcomes after esophagectomy: a systematic review. Ann Surg.

[9] Kingma BF, Steenhagen E, Ruurda JP, van Hillegersberg R (2017). Nutritional aspects of enhanced recovery after esophagectomy with gastric conduit reconstruction. J Surg Oncol.

[10] Koyanagi K, Tachimori Y (2017). Early oral nutrition plays an active role in enhanced recovery after minimally invasive esophagectomy. J Thorac Dis.

[11] Braga M, Gianotti L, Gentilini O, Parisi V, Salis C, di Carlo V (2001). Early postoperative enteral nutrition improves gut oxygenation and reduces costs compared with total parenteral nutrition. Crit Care Med.

[12] Han H, Pan M, Tao Y, Liu R, Huang Z, Piccolo K, Zhong C, Liu R (2018). Early enteral nutrition is associated with faster post-esophagectomy recovery in Chinese esophageal cancer patients: a retrospective cohort study. Nutr Cancer.

[13] Lorimer PD, Motz BM, Watson M, Trufan SJ, Prabhu RS, Hill JS, Salo JC (2019). Enteral feeding access has an impact on outcomes for patients with esophageal cancer undergoing esophagectomy: an analysis of SEER-Medicare. Ann Surg Oncol.

[14] Berkelmans GH, van Workum F, Weijs TJ (2017). The feeding route after esophagectomy: a review of literature. J Thorac Dis.

[15] Sethuraman SA, Dhar VK, Habib DA, Sussman JE, Ahmad SA, Shah SA, Tsuei BJ, Sussman JJ, Abbott DE (2017). Tube feed necrosis after major gastrointestinal oncologic surgery: institutional lessons and a review of the literature. J Gastrointest Surg Off J Soc Surg Aliment Tract.

[16] Afaneh C, Gerszberg D, Slattery E, Seres DS, Chabot JA, Kluger MD (2015). Pancreatic cancer surgery and nutrition management: a review of the current literature. Hepatobiliary Surg Nutr.

[17] Dholaria S, Lakhera KK, Patni S (2017). Intussusception: a rare complication after feeding jejunostomy; a case report. Indian J Surg Oncol.

[18] Linder G, Lindblad M, Djerf P, Elbe P, Johansson J, Lundell L, Hedberg J (2016). Validation of data quality in the Swedish National Register for Oesophageal and Gastric Cancer. Br J Surg.

[19] Ludvigsson JF, Almqvist C, Edstedt Bonamy A-K (2017). Registers of the Swedish total population and their use in medical research. Eur J Epidemiol.

[20] Klevebro F, Johar A, Lagergren J, Lagergren P (2018) Outcomes of nutritional jejunostomy in the curative treatment of esophageal cancer. Dis Esophagus Off J Int Soc Dis Esophagus. 10.1093/dote/doy113 10.1093/dote/doy113 30496419

[21] Dalton BGA, Friedant AJ, Su S, Schatz TAP, Ruth KJ, Scott WJ (2017). Benefits of supplemental jejunostomy tube feeding during neoadjuvant therapy in patients with locally advanced, potentially resectable esophageal cancer. J Laparoendosc Adv Surg Tech A.

[22] Koterazawa Y, Oshikiri T, Hasegawa H, Yamamoto M, Kanaji S, Yamashita K, Matsuda T, Nakamura T, Suzuki S, Kakeji Y (2019) Routine placement of feeding jejunostomy tube during esophagectomy increases postoperative complications and does not improve postoperative malnutrition. Dis Esophagus Off J Int Soc Dis Esophagus. 10.1093/dote/doz021 10.1093/dote/doz021 30997494

[23] Weijs TJ, van Eden HWJ, Ruurda JP (2017). Routine jejunostomy tube feeding following esophagectomy. J Thorac Dis.

[24] Jenkins TK, Lopez AN, Sarosi GA, Ben-David K, Thomas RM (2018). Preoperative enteral access is not necessary prior to multimodality treatment of esophageal cancer. Surgery.

